# Results of Gensingen Bracing in Patients With Adolescent Idiopathic Scoliosis: Retrospective Cross-Sectional Feasibility Study

**DOI:** 10.2196/50299

**Published:** 2024-01-10

**Authors:** Xiaofeng Nan, Tuğba Kuru Çolak, Burçin Akçay, Hua Xie, Liwei Zhao, Maksym Borysov

**Affiliations:** 1 Nan Xiaofeng's Spinal Orthopedic Workshop Xi'an Shaanxi China; 2 Department of Physiotherapy and Rehabilitation Faculty of Health Sciences Marmara University İstanbul Turkey; 3 Department of Physiotherapy and Rehabilitation Faculty of Health Sciences Bandırma Onyedi Eylül University Balıkesir Turkey; 4 Schroth Health Technology Chongqing China; 5 National Research Centre for Rehabilitation Technical Aids, Schroth Health Technology Beijing China; 6 Orttech Plus Rehabilitation Service Kharkiv Ukraine

**Keywords:** scoliosis, brace treatment, feasibility study, outcome, skeletal, spine, back, musculoskeletal, curvature, spinal, database, template, design, brace, orthopedics, injury, rehabilitation, Gensingen brace, conservative brace treatment, Idiopathic Scoliosis, orthopedic, injuries, data science, data management

## Abstract

**Background:**

Bracing is an essential part of scoliosis treatment. The standard of brace treatment for patients with scoliosis today is still very variable in terms of brace quality and outcome. The Gensingen brace is a further developed Chêneau brace derivative with individual design, which can be adapted through computer-aided design.

**Objective:**

This study aims to generate a template to obtain a database for prospective multicenter studies study to analyze the results of high-corrective asymmetric Gensingen brace treatment for patients with adolescent idiopathic scoliosis (AIS).

**Methods:**

A template for the database was created, which contains the patients' basic data (age, menarcheal status, Risser Sign, curve pattern, and daily brace wearing time), the Cobb angles of curvature, and the cosmetically relevant angles of trunk rotation (ATR). A retrospective review of medical records of patients with AIS, who met the Scoliosis Research Society’s inclusion criteria for brace studies, was performed to test the feasibility of the template. Template items were filled in by the researchers.

**Results:**

Out of 115 patients between 2014 and 2018, the complete data of 33 patients followed up at least 3 months after complete Gensingen brace weaning could be analyzed. The mean age was 12 years, the mean Cobb angle was 33.6°, and the mean Risser value was 0.7 at the beginning of the treatment. The mean improvement in the Cobb angle on in-brace x-ray imaging was –26.1^०^ (80% of in-brace correction). The Cobb angle of the major curvature changed as follows: curve stabilization was achieved in 7 (21.2%) cases, and curve improvement was achieved in 26 (78.8%) cases. None of the patients showed a curve progression. The Cobb angle was significantly reduced in the brace at the end of treatment and at follow-up evaluation *(P*<.001). ATR improved significantly for thoracic (*P*<.001) and lumbar curves (*P*<.001).

**Conclusions:**

The database proved to be informative in the assessment of radiological and clinical outcome parameters. The example data set we have generated can be a helpful tool for professionals who work in clinics but do not store regular patient data. Especially with regard to different patient collectives worldwide, different results may be achieved with the same standards of care. In addition, the results of this study suggest that above-average correction effects with a full-time brace application lead to significant improvements in the Cobb angle after brace treatment has been completed.

## Introduction

3D spinal deformities, called scoliosis, can have different causes. What most forms of scoliosis have in common is that they tend to progress in curvature during periods of increased growth. In most cases (between 80% and 90%), scoliosis affects otherwise healthy individuals and first appears during the pubertal growth spurt [1-4].

Treatment of adolescent idiopathic scoliosis (AIS) consists of corrective exercise treatments, the application of various braces, and surgical treatment [5]. High-quality studies support the use of physical therapy measures [6-8] and brace application [9-13].

Scoliosis can progress rapidly, especially in adolescence—a period of rapid growth. Therefore, it is very important to apply evidence-based treatment approaches promptly. When patients are meaningfully “observed” rather than braced, a curve progression of 6° within a period of 6 months is between 20% and 40% more likely in growing children and adolescents [1]. Hence, it is crucial that patients with AIS receive conservative management treatments as soon as possible after their diagnosis, especially if they are premenarchal and still have significant growth potential [14].

Despite the existing evidence for treatment with braces, there is a significant variation in the success rates of different brace applications and even within individual brace families. Meanwhile, it is crystallizing that highly corrective asymmetric braces are superior to a more symmetrically compressive thoracolumbosacral orthosis. However, even with asymmetric brace applications, the quality of treatment is highly variable [15]. Therefore, to ensure patient safety, only computer-aided design (CAD) brace series should be used, which are subject to a quality management program and that use standardized adjustment algorithms corresponding to the curvature pattern [15-17].

One of these brace series is the Gensingen Brace (GBW) [18,19], used in our centers and other centers worldwide. Based on our clinical experience, we hypothesize that the progression of curvature in children with AIS treated with GBW can be stopped and that there would be improvements in curvature in a certain proportion of the cohort [19,20].

Although GBW efficacy has been demonstrated in previous studies published in the literature, follow-up studies after completion of treatment are limited [19,20].

The purpose of this study is to test the feasibility of a prospective multicenter study by generating a database, including radiological and clinical outcome parameters. For this purpose, the database has been tested with a retrospective review of medical records of patients from 1 center.

## Methods

### Ethical Considerations

This retrospective cross-sectional study was conducted in accordance with the tenets of the Declaration of Helsinki. Ethics approval for the study was obtained from the Ethics Committee of Bandırma University (2022/195). The parents of each child were informed of the study procedures, and written consent of the caregivers and participants was obtained which in accordance with the ethics committee’s guidelines. The data set did not contain any identifiable information.

### Study Design

This paper reports the results of treatment with a GBW for AIS in a retrospective nonrandomized feasibility study.

### Recruitment

Patients who were admitted to Nan Xiaofeng's Spinal Orthopedic Workshop and Schroth Health Technology centers between 2014 and 2018 and were treated with a GBW and followed up at least 3 months after complete brace weaning were included in this study.

A template for the database to be tested was created, which contains the basic data of the patients and their Cobb curvature angles and the cosmetically relevant angles of trunk rotation (ATR). A retrospective review of medical records of patients with AIS, who met the Scoliosis Research Society’s (SRS’s) inclusion criteria for brace studies [20], was performed, and the investigators then filled in the template. These criteria were as follows: female patients with prescribed brace treatment for AIS, aged between 10 and 14 years, with a Cobb angle between 25° and 40° for at least 1 structural curve, during growth with a Risser stage between 0 and 2, premenarcheal or less than 1 year after menarche, and without previous treatment [21].

Patients with nonidiopathic scoliosis; other orthopedic, neuromuscular, or rheumatic diseases; mental or psychiatric problems; iliac crest ossification of Risser stage 3-5, or continuing treatment were excluded.

According to the current guidelines, it is recommended that patients with Risser stage 0-3 and a scoliosis progression risk of more than 60% according to the Lonstein and Carlson [22] formula should start bracing treatment. In this study, risk of progression was calculated and brace treatment was recommended to the patients. For brace treatment to be effective, full-time use was recommended [23].

**Figure 1:**
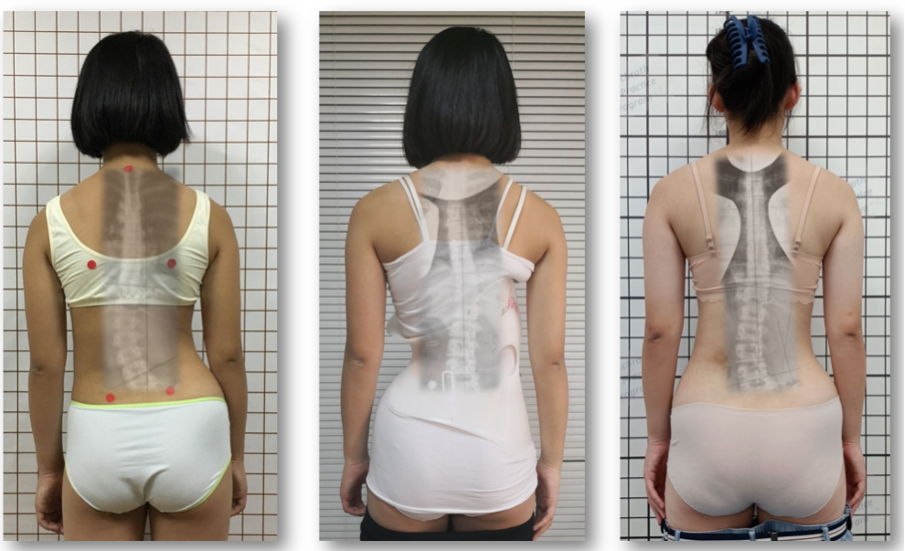
A 12-year-old minor patient with a single lumbar curve of 32° treated with a short Gensingen brace (GBW) with full in-brace correction (middle picture). Final outcome 12 months after brace weaning with a curvature of 22° with a nicely recompensated clinical appearance (right).

**Figure 2:**
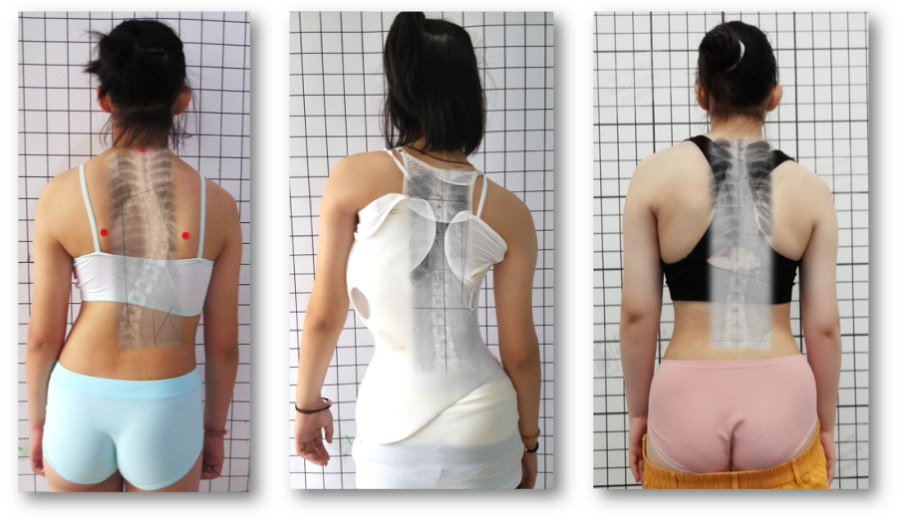
A 12-year-old minor patient with a single thoracic curve of 48° treated with a functional 3-curve balanced with a minor and shorter lumbar countercurve and Gensingen brace (GBW) with full in-brace correction (middle picture). Final outcome 9 months after brace weaning with a curvature of 28° with a nicely recompensated clinical appearance (right).

**Figure 3:**
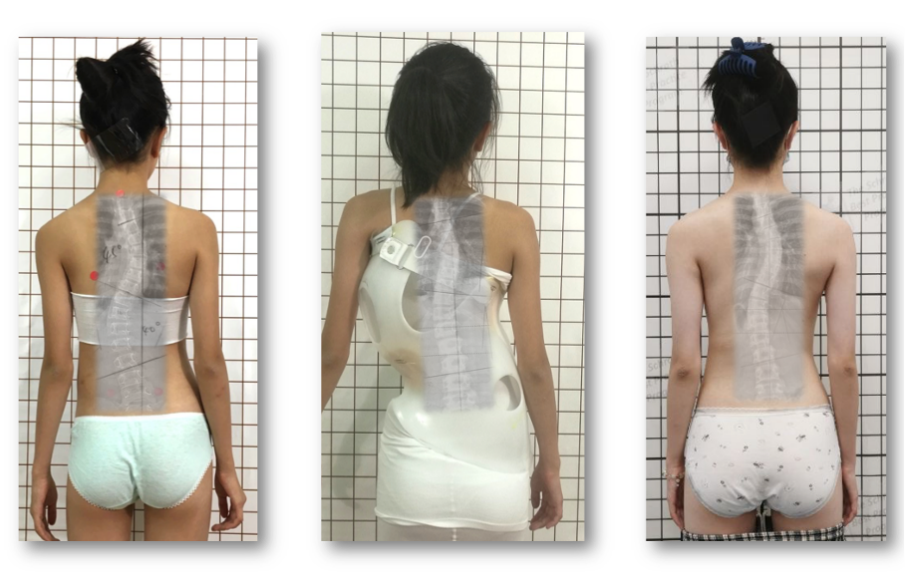
A 12-year-old minor patient with a Lenke 6 combined curve of 45° (thoracic) and 40° (lumbar) treated with a functional 4-curve, double curvature and Lenke Gensingen brace (GBW) with good in-brace correction (middle picture). Final outcome 15 months after brace weaning and a curvature of 37° (thoracic) and 32° (lumbar) with a nicely recompensated clinical appearance (right). In particular, a Lenke 6 pattern is not as easy to correct with a brace like other curve patterns.

All children in this study used the GBW (Figures 1-3).

The GBW is a further developed Chêneau brace derivative with individual design, which can be adapted through CAD. Customization, accuracy, and quality control of scoliosis braces are significantly aided by CAD. By using this technology, braces can be generated specifically for each patient's particular spinal curve pattern, resulting in more effective and comfortable treatment. The individual production steps have already been described in the literature [18]. First, the patient is scanned, and patient data are collected and entered into the database together with the x-ray image. Based on these data, the basic model corresponding to the curvature pattern is first selected from the brace library.

The patient’s scan is cropped and scaled. Then, the selected brace is inserted into the scene and adjusted in accordance with the individual’s body shape. Then, the correction algorithms specified for the particular pattern and curvature strength (Cobb angle) are applied accordingly. The result is a brace model that reflects the respective curvature pattern and the individual entities of the patient [24].

The following brace weaning process was applied. For curves with an initial curve grade of ≤35, the brace wearing time was decreased by wearing the brace for 16 hours per day for 3 months, 12 hours per day for 3 months, and at night for 6 months. For curves above the initial grade of 35, brace treatment was terminated by wearing a brace for 16 hours per day for 12 months, 12 hours per day for 12 months, and 6 months at night.

### Database Template

The template for the database contained the following: the patient's age (in years) before starting treatment and the menarcheal status (in months) were recorded. Risser's sign and curvature pattern, according to the Augmented Lehnert-Schroth (ALS) classification, were evaluated on pretreatment x-ray imaging. The Cobb angle and ATR were evaluated as primary outcome measures. The progression factor was calculated with the Cobb angle, patient's age, and Risser's finding. Daily brace-wearing time was recorded by asking parents and patients.

Risser's sign determines bone maturity, growth rate, and progression risk of a patient with scoliosis. It has been reported to be reliable and sensitive in determining bone maturity. Risser grading was assessed on the anteroposterior radiograph. The epiphyseal plate starts becoming visible from the lateral edge of the anterior superior iliac spine, progresses medially, and finally fuses at the posterior superior iliac spine. Degree of completion was indicated as a percentage: grade 1: ≤25%; grade 2: between 26% and 50%; grade 3: between 51% and 75%; and grade 4: between 75% and 100%. When the epiphyseal plate is fully fused to the ilium, it is defined as being grade 5 [25].

Curve classification was performed in accordance with the ALS classification that was developed as an expansion of the Lehnert-Schroth classification and included eight different curvature types: (1) 3CH: functional 3-curve, with hip prominence; (2) 3CTL: functional 3-curve, thoracolumbar, which implies a functional 3-curve with hip prominence and a thoracolumbar apex at thoracic vertebra 12; (3) 3C: functional 3-curve balanced with a minor and shorter lumbar countercurve; (4) 3CL: functional 3-curve lumbar with a long lumbar countercurve; (5) 4C: functional 4-curve, double curvature; (7) 4CL: functional 4-curve with major lumbar curvature; and (8) 4CTL: functional 4-curve with major thoracolumbar curvature (and an apex at lumbar vertebra 1) [26].

The Cobb method was used to measure the degree of curvature: vertical lines were drawn on the superior and inferior vertebral endplate lines of the neutral vertebrae on the anteroposterior x-ray image of the whole spine [27], and the angle of the 2 vertical lines was recorded. X-ray images were taken at four stages: (1) before treatment (baseline), (2) at 4 to 6 weeks after the brace was fitted (in-brace), (3) at the end of treatment, and (4) at follow-up assessment after brace weaning. All braceless x-ray images were taken at least 24 hours after removal to eliminate the brace effect. All x-ray measurements were taken independently by the same experienced orthopedist. The difference between the Cobb angle at follow-up and that before treatment were calculated. Based on this difference, 3 possible outcomes are distinguished in accordance with the International Society On Scoliosis Orthopaedic and Rehabilitation Treatment’s guidelines: curve correction (≤–5° Cobb angle), curve stabilization (>–5° and <5° Cobb angle), and curve progression (≥5° Cobb angle) [23].

The ATR is the most commonly used method for clinical and cosmetic assessment of scoliosis. ATR of 86% repeatability is supposed to be a reliable measurement. A change of 2° in interobserver measurements is considered significant [28]. ATR are measured using a special inclinometer called a scoliometer (according to Bunnel [28]). The patient was asked to bend forward with relaxed arms (Adam’s forward bend test). The scoliometer is placed on the back of the patients, and the maximum degree of each curve was recorded [28]. ATR measurements obtained before treatment and at follow-up assessment were analyzed.

The risk for progression of the Cobb angle was calculated using the progression factor formula in accordance with Lonstein and Carlson [22]:


Risk for Cobb angle progression = Cobb angle – (3 × Risser stage) / chronological age (in years) (1)

The International Society On Scoliosis Orthopaedic and Rehabilitation Treatment’s guidelines and the validated Schroth Best Practice Academy Guidelines suggest using this formula to decide treatment indications and avoid over- and undertreatment [29,30].

According to this formula, observation is recommended for cases with a risk factor of 1.4 and below (<40% incidence of progression), physiotherapy is recommended for cases with a risk factor of 1.4-1.6 (between 40% and 60% incidence of progression), and brace treatment is recommended for cases with a risk factor of 1.6 and above (>60% incidence of progression) [31].

### Statistical Analysis

Data analysis was performed using SPSS (version 16; IBM Corp). The Shapiro-Wilk test was used to test the normality of each variable. *P* values less than .05 were considered statistically significant for a 2-tailed test. Mean (SD) values and minimum and maximum values were determined using descriptive statistics.

Repeated-measures ANOVA was used to compare Cobb angle values at baseline, in-brace, end of treatment, and follow-up, and a paired samples *t* test was used to compare ATR values at baseline and follow-up.

## Results

Out of 115 patients from 2014 to 2018, complete data of 33 patients who could be followed up at least 3 months after complete brace weaning have been analyzed. The mean age was 12 years, the mean Cobb angle was 33.6°, and the mean Risser value was 0.7 at the beginning of the treatment (Table 1). Based on the ALS classification, most cases (45.5%) had a 3C scoliosis pattern (major thoracic curve). A total of 18 of the patients were premenarcheal, and menarche had started in 15 patients (mean 5.7 months).

**Table 1:** Baseline demographic and clinical characteristics of patients.

Variables		Value
Age (years), mean (SD; range)		12 (1.06; 10-14)
Risser value, mean (SD; range)		0.7 (0.8; 0-2)
Main Cobb angle (°),mean (SD; range)		33.6 (8.1; 22-50)
Angle of trunk rotation (°; thoracic), mean (SD; range)		9.4 (5.1; 2-21)
Angle of trunk rotation (°; lumbar), mean (SD; range)		5.5 (4.05; 0-15)
**Main curve location, n (%)**		
	Thoracic	26 (78.8)
	Lumbar	7 (21.2)
**Augmented Lehnert-Schroth curve classification, n (%)**		
	3CH^a^	5 (15.2)
	3CL^b^	15 (45.5)
	3CN^c^	5 (15.2)
	4C^d^	6 (18.2)
	4CTL^e^	2 (6.1)

^a^3CH: functional 3-curve, with hip prominence.

^b^3CL: functional 3-curve lumbar with a long lumbar countercurve.

^c^3CN: functional 3-curve, compensated.

^d^4C: functional 4-curve, double curvature.

^e^4CTL: functional 4-curve with major thoracolumbar curvature.

The mean treatment period with the brace was 33.6 (SD 10.1, range 15-51) months, and the mean follow-up duration was 12 (SD 6.1, range 3-35) months. Daily brace wearing time in the first year of the brace treatment was 21.3 (SD 1.2, range 16-22) hours. All patients reported wearing the brace for at least 20 hours each day, with the exception of 1 who only wore it for 16 hours.

**Figure 4:**
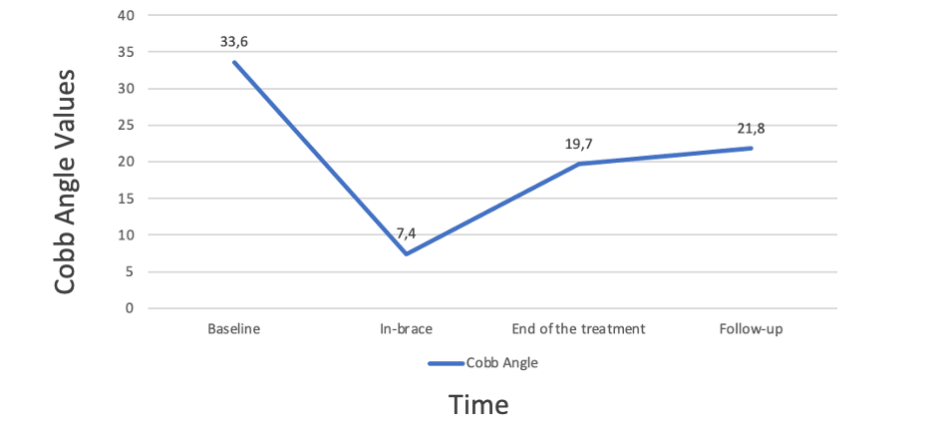
Changes in the main Cobb angle over time.

The mean improvement in Cobb angle on x-ray imaging performed in the brace was –26.1° (SD 6.8°, range –43° to –12°; Figure 4), which implies a correction effect in the brace of 80%. The difference in Cobb angle at baseline and follow-up was –11.7° (SD 6.8°, range –24° to 0°; a 35% improvement from the initial value). The change in ATR at baseline and follow-up was –4.5° (SD 4.5°, range –13° to –6°; a 49% improvement from the initial value), and the change in lumbar ATR was –3.2° (SD 4.2°, range –12° to –7°; a 62% improvement from the initial value). Changes in the Cobb angle and thoracic and lumbar ATR values at the end of treatment were significant (Table 2).

**Table 2:** Changes in the Cobb angle and angles of trunk rotation (ATR).

Outcome measurements		Value, mean (SD; range)		*P* value
**Main Cobb angle (°)**				
	Baseline	33.6 (8.1; 22 to 50)	<.001^a^	
	In-brace	7.4 (7.9; –11 to 25)	<.001^b^	
	End of treatment	19.7 (9.3; 2 to 42)	<.001^b^	
	Follow-up	21.8 (9.2; 3 to 42)	<.001^b^	
**Thoracic ATR (°)**				<.001
	Baseline	9.4 (5.1; 2 to 21)		
	Follow-up	4.4 (2.6; –2 to 12)		
**Lumbar ATR (°)**				<.001
	Baseline	5.5 (4.05; 0 to 15)		
	Follow-up	2.3 (2.3; –3 to 8)		

^a^Repeated-measures ANOVA.

^b^Paired samples *t* test.

The mean progression risk factor was 2.6 (SD 0.7, range 1.43-4.55), which, in the case of untreated scoliosis, would correspond to a probability of progression of far more than 95% reported by Lonstein and Carlson [22]. According to the SRS’s criteria, curve stabilization was achieved in 7 (21.2%) cases, and curve improvement was achieved in 26 (78.8%) cases. None of the patients had a curve progression in this sample with a probability of progression of far more than 95% reported by Lonstein and Carlson [22].

The improvement in the Cobb angle achieved in the brace was negatively moderately correlated with the pretreatment Cobb angle (*P*=.008, *r*=–0.452). There was a positive moderate correlation between the amount of change in Cobb angle obtained at the end of treatment and the amount of improvement obtained in the brace (*P*<.001, *r*=0.593).

## Discussion

### Principal Findings

Our study shows that the template generated can be used for future prospective multicenter studies. On analyzing the data we saved in the template, the results showed that the GBW, which provides a 3D correction, is effective in stopping curvature progression and reducing the angle of curvature in adolescents with idiopathic scoliosis who continue to experience vertebral growth and are at high risk of progression.

Brace treatment and scoliosis-specific exercise methods are the most widely used, accepted, and effective treatment methods for patients with AIS [6-11,31,32]. Extensive evidence in the literature shows the effectiveness of brace treatment [15,33,34]. Previous studies have reported that brace treatment stops progression, corrects moderate curves, and reduces the rate of surgical indication [33-35]. Our results show that besides stopping curvature progression with high-correction full-time bracing also potentially improves the Cobb angle and ATR.

After the onset of the initial deformity, it is generally accepted that AIS progresses with asymmetric vertebral growth that occurs during the growth spurt. Adolescence is one of the periods of rapid growth. It has been reported that children with a high risk for progression during the rapid growth period experience progression in their curvature when left untreated [31].

In this study, the risk of progression was >95%, according to the formula developed by Lonstein and Carlson [22]. However, when growth was complete and in subsequent evaluations, it was found that there was no progression at all. The Cobb angle did not increase by ≥5° in any patient.

The patient population included in this study does not differ significantly from the cohorts of previously published studies in terms of age, maturity, menarcheal status, Cobb angle, and curvature pattern distribution [18,19].

Weiss et al [19] assessed 28 patients with AIS with a mean age of 12.7 years and Cobb angle of 30.5° using the GBW. However, they carried out their final evaluation an average of 24 months after brace treatment was initiated. They reported that the in-brace correction in their sample was from 33.9° to 15.9°, indicating an average correction of 52.7%.

In another study, Weiss et al [18] observed 167 patients with AIS who were treated with a GBW over a period of at least 18 months. The authors reported a 47%-52% rate of correction of the Cobb angle of the main curve in the brace [18]. When we calculated the success rate in accordance with the Cobb angle obtained in the brace, the treatment success rate was 80% in our cases.

In previous studies [18,19], the success rate at the end of treatment was between 86% and 92% in different subcohorts, but in our study, progression in curvature was stopped and no longer observed in all children. Therefore, GBW’s success may be considered as 100% in this study. Since the brace design worldwide follows standardized CAD algorithms and the material (high-density polyethylene) does not differ from that used in other studies, the specifics of the studied collective might play a role. The cohort studied is from mainland China, and it is possible that the patients included in this study take brace treatment more seriously than may be the case in other countries. Another factor may be that brace treatment in China has to be financed by the patients or their parents themselves, which may also improve their motivation to wear the brace.

The main curvature Cobb angle at first diagnosis was >40° in 8 children included in this study. Considering that the Risser grade is low and the growth potential of these children is high, it is predicted that the curvatures will most likely progress. However, children with a curvature of >40° completed their treatment with an average of 16.7° (range 2°-34°). Based on these results, the use of GBWs significantly reduces the need for surgical treatment in children with AIS.

In this study, a template prepared by the investigators was filled with the help of a retrospective review of medical records. Our study shows that it would be appropriate to use this template in future prospective studies and the data intended to be recorded in this template can indicate treatment effectiveness for brace treatment. An international multicenter study considering the SRS’s inclusion criteria for brace treatment studies seems feasible.

Our study supports the conclusions of other studies regarding the corrective effect of the brace [36,37] and confirms previous findings in this field, which show that above-average corrective effects with full-time brace application lead to significant improvements in the Cobb angle after completion of brace treatment [38,39].

Evaluation of the treatment outcomes with the Cobb angle, which is still accepted as the gold standard today, the establishment of the study sample group considering the SRS’s brace study criteria, and continuation of the follow-up of the children after the end of treatment can be considered as the strengths of the study.

### Limitations

The study’s limitations include our inability to determine the changes specific to different curve patterns, the fact that the effectiveness of the brace was not evaluated at different daily wearing times, and the fact that daily brace wearing time was recorded in accordance with the participants’ families statement. We suggest investigating the effectiveness of brace treatment in different curvature patterns and different wearing times with larger sample groups in future studies.

### Conclusions

The results of this study suggest that above-average correction effects with full-time brace application lead to significant improvements of the Cobb angle upon completion of brace treatment. The example data set we have generated can be a helpful tool for professionals who work in clinics but do not store regular patient data. Furthermore, prospective multicentral studies with large samples can be conducted by collecting the same data at different centers.

## Abbreviations

3C: functional 3-curve balanced with a minor and shorter lumbar countercurve
3CH: functional 3-curve, with hip prominence
3CL: functional 3-curve lumbar with a long lumbar countercurve
3CTL: functional 3-curve, thoracolumbar
4C: functional 4-curve, double curvature
4CL: functional 4-curve with major lumbar curvature
4CTL: functional 4-curve with major thoracolumbar curvature
AIS: adolescent idiopathic scoliosis
ALS: Augmented Lehnert-Schroth
ATR: angles of trunk rotation
CAD: computer-aided design
GBW: Gensingen Brace
SRS: Scoliosis Research Society

## Data Availability

The data that support the findings of this study are available upon request from the authors.
